# Neural ordinary differential equations with irregular and noisy data

**DOI:** 10.1098/rsos.221475

**Published:** 2023-07-19

**Authors:** Pawan Goyal, Peter Benner

**Affiliations:** Max Planck Institute for Dynamics of Complex Technical Systems, Standtorstrasse 1, 39106 Magdeburg, Germany

**Keywords:** machine learning, dynamical systems, neural networks, noisy data, neural ordinary differential equations

## Abstract

Measurement noise is an integral part of collecting data of a physical process. Thus, noise removal is necessary to draw conclusions from these data, and it often becomes essential to construct dynamical models using these data. We discuss a methodology to learn differential equation(s) using noisy and irregularly sampled measurements. In our methodology, the main innovation can be seen in the integration of deep neural networks with the neural ordinary differential equations (ODEs) approach. Precisely, we aim at learning a neural network that provides (approximately) an implicit representation of the data and an additional neural network that models the vector fields of the dependent variables. We combine these two networks by constraints using neural ODEs. The proposed framework to learn a model describing the vector field is highly effective under noisy measurements. The approach can handle scenarios where dependent variables are unavailable at the same temporal grid. Moreover, a particular structure, e.g. second order with respect to time, can easily be incorporated. We demonstrate the effectiveness of the proposed method for learning models using data obtained from various differential equations and present a comparison with the neural ODE method that does not make any special treatment to noise. Additionally, we discuss an ensemble approach to improve the performance of the proposed approach further.

## 1. Introduction

Uncovering dynamical models explaining physical phenomena and dynamic behaviours has been active research for centuries. When a model describing the underlying dynamics is available, it can be used for several engineering studies such as process design, optimization, predictions and control. Conventional approaches based on physical laws and empirical knowledge are often used to derive dynamical models. However, this is impenetrable for many complex systems, e.g. understanding the Arctic ice pack dynamics, sea ice, power grids, neuroscience or finance, to only name a few applications. Data-driven methods to discover models have enormous potential to better understand transient behaviours in the latter cases. Furthermore, data acquired using imaging devices or sensors are contaminated with measurement noise. Therefore, systematic approaches to learning dynamical models with proper noise treatment are required.

In this work, we consider learning autonomous nonlinear differential equation of the form

1.1 $$\dot{x}(t)=g(x(t))\;\mathrm{and}\;x(0)=x_{0},$$

where $x(t)\in R^{n}$ denotes the solution at time *t*, $\dot{x}(t)$ is the time-derivative of **x** at time *t*, and the continuous function $g(\cdot )\;:\;R^{n}\to R^{n}$ defines the vector field. We aim to learn to the vector field **g**(·) using the noisy measurements. Towards this aim, the initial work [1] proposes a framework that explicitly incorporates the noise into a numerical time-stepping method, namely a *Runge–Kutta* method. Though the approach has shown promising directions, its scalability remains ambiguous as the approach explicitly needs noise estimates and aims to decompose the signal explicitly into noise and ground truth. Moreover, it requires that the Runge–Kutta method can give a reasonable estimate at the next step. Additionally, irregular sampling (e.g. when dependent variables are not collected or not available at the same time grid) cannot be applied, which can be highly relevant when information is gathered from various sources, e.g. in medical applications. This work discusses a deep learning-based approach to learning dynamical models by enhancing neural networks with adaptive numerical integrations. This allows learning models to represent the vector field accurately without estimating noise explicitly and when dependent variables are arbitrarily irregularly sampled.

### 1.1. Our contributions

**Figure 1.**
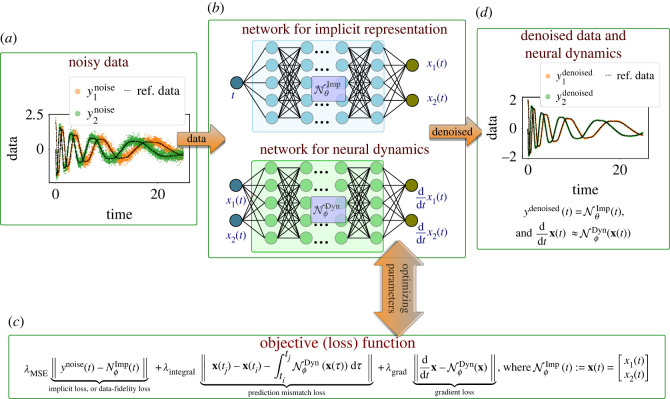
The figure illustrates the framework for denoising the data and learning a model describing underlying dynamics. For this, we determine an implicit representation of the noisy data (approximately) by a network $N_{\theta }^{\mathrm{Imp}}$ and another network for the vector field $N_{\phi }^{\mathrm{Dyn}}$. These two networks are connected by enforcing that the dynamics of the output of the implicit representation can be given by $N_{\phi }^{\mathrm{Dyn}}$. Once the objective function (shown in *c*) is minimized, we obtain an implicit network for denoised data and a model for the vector field $N_{\phi }^{\mathrm{Dyn}}(x)$.

Our work introduces a framework to learn dynamical models by innovatively blending neural networks and numerical integration methods from noisy and irregular measurements. Precisely, we aim at learning two networks: one that approximately represents the given measurement data implicitly, and the second one that approximates the vector field. We connect these two networks by enforcing an integral form of the ordinary differential equation (ODE) as depicted in figure 1. The appeal of the approach is that we do not require an explicit noise estimate to learn a model. Furthermore, the proposed approach is applicable even if each dependent variable is collected on a different time grid, which can be irregular.

The remaining structure of the paper is as follows. In the next section, we present a summary of relevant work. In §3, we present our deep learning-based framework for learning dynamics from noisy measurements by combining two networks. In §4, we also demonstrate the effectiveness of the proposed methodology using various synthetic data with increasing noise levels. Section 5 discusses the application of learning second-order dynamical models. Moreover, in §6, we discuss how to handle irregular sampling of measurements. We conclude the paper with a summary and future research directions. We also discuss an ensemble approach [2–6] to improve our approach further by taking a mean of the ensemble models.

## 2. Relevant work

Data-driven methods to learn dynamical models have been studied for several decades (e.g. [7–9]). Learning linear models from input–output data goes back to Ho & Kálmán [10]. There have been several algorithmic developments for linear systems, for example, the eigensystem realization algorithm [11,12], and Kalman filter-based approaches [13–15]. Dynamic mode decomposition has also emerged as a promising approach to construct models from input–output data and has been widely applied in fluid dynamics applications (e.g. [16–18]). Furthermore, there has been a series of developments to learn nonlinear dynamical models. This includes, for example, equation-free modelling [19], nonlinear regression [20], dynamical modelling [21] and automated inference of dynamics [22–24]. Using symbolic regression and an evolutionary algorithm [25,26], learning compact nonlinear models becomes possible. Moreover, leveraging sparsity (also known as sparse regression), several approaches have been proposed [27–32]. We also mention the work [33] that learns models using Gaussian process regression. All these methods have particular approaches to handling noise in the data. For example, sparse regression methods (e.g. [27,28,32]) often use smoothing methods before identifying models, and the work [33] handles measurement noise as data represented like a Gaussian process.

Even though the aforementioned nonlinear modelling methods are appealing and powerful in providing analytic expressions for models, they are often built upon model hypotheses. For example, the success of sparse regression techniques relies on the fact that the nonlinear basis functions, describing the dynamics, lie in a candidate feature library. For many complex dynamics, the utilization of these methods is not trivial. Thus, machine learning techniques, particularly deep learning-based ones, have emerged as powerful methods capable of expressing any complex function in a black-box manner given enough training data. Neural network-based approaches in the context of dynamical systems have been discussed in [34–37] decades ago. A particular type of neural network, namely recurrent neural networks, intrinsically models sequences and is often used for forecasting [38–42] but does not explicitly learn the corresponding vector field. Deep learning is also used to identify a coordinate transformation so that the dynamics in the transformed coordinates are almost linear or sparse in a high-dimensional feature basis (e.g. [43–46]). Furthermore, we mention that classical numerical schemes are incorporated with feed-forward neural networks to have discrete-time steppers for predictions (see [36,47–49]). The approaches in [36,47] can be interpreted as nonlinear autoregressive models [9]. A crucial feature of deep learning-based approaches that integrate numerical integration schemes is that vector fields are estimated using neural networks. Also, time-stepping is done using a numerical integration scheme. Furthermore, in recent times, *neural ordinary differential equations* (neural ODEs) in which neural networks define the vector fields, have been proposed in [50], where it is shown how to compute gradients with respect to network parameters efficiently using adjoint sensitivities. As a result, one can use efficient black-box numerical solvers to solve ODEs in a given time span using any adaptive time-stepping method. However, measurement data are often corrupted with noise, and these approaches do not perform any specific noise treatment. The work in [1] proposes a framework that explicitly incorporates the noise into a numerical time-stepping method. Though the approach has shown a promising direction, its scalability remains ambiguous. The approach explicitly needs noise estimates by learning the decomposition of the signal into noise and ground truth. Also, it relies on a Runge–Kutta scheme that can accurately estimate the variable at the next step. In the context of sparse regression, several attempts have been made to reduce the effect of the noise on the discovered sparse models, which are, for example, WSINDy [51] and Ensemble-SINDy [52]. However, these techniques rely on sparse regression assumptions and assume all dependent variables are collected at the same time. Furthermore, in scenarios where the data are collected on an irregular time grid, the work [53] discussed a methodology by combining gated recurrent unit (GRU) and neural ODEs. In the approach, an estimate for the initial condition of (latent) ODEs is learned, and an ODE for the vector field is then integrated using the estimated initial condition. However, long sequences are quite challenging to estimate the initial condition given measurements future in time. Although in [53] the measurements can be collected at an irregular time grid, it still requires that all dependent variables are measured at the same time grid. When each dependent variable is collected at a different time grid, the approach [53] is not even applicable. Gaussian processes have recently been combined with neural ODEs to deal with noisy measurements and irregular measurement sampling [54]. In this, each dependent variable is represented as a Gaussian process, and a probabilistic model is learned, describing the underlying dynamics. The approach, however, depends on the modelling assumption for each dependent variable and yields a probabilistic model rather than a deterministic model. Furthermore, it does not focus on recovering the clean data from the noisy measurements.

## 3. Proposed methodology for learning dynamics: implicit networks combined with neural ODEs

This section discusses our framework for learning dynamical models using noisy measurements without explicit noise estimation. To achieve the goal, we use the powerful approximation capabilities of deep neural networks and their automatic differentiation feature with the neural ODEs approach [50]. Neural ODEs allow one to integrate a function, defining the vector field, with any desired method and accuracy, and computing derivatives with respect to the parameters efficiently. For details, refer to Chen *et al.* [50]. Consider the nonlinear dynamical system of the form (1.1). Note that the solution **x**(*t*_*j*_) can be given as

3.1 $$x(t_{\;j})=x(t_{i})+\int_{t_{i}}^{t_{\;j}}g(x(\tau ))\;d\tau .$$

Next, we discuss our framework to learn dynamical models from noisy measurements. The approach involves two networks. The first network implicitly represents the variable as shown in figure 1*b*, and the second network approximates the vector field, or the function **g**(·). These two networks are related by connecting the dependent variables at time *t*_*i*_ and *t*_*j*_, as given in (3.1). That is, the output of the implicit network is not only in the vicinity of the given noisy measurement data, but also its time-evolution can be defined by **g**(**x**) or as in (3.1).

To be mathematically precise, let us denote noisy measurement data at time *t*_*i*_ by $y(t_{i})$. Furthermore, we consider a feed-forward neural network, denoted by $N_{\theta }^{\mathrm{Imp}}$ and parameterized by ***θ***, that approximately yields an implicit representation of measurement data, i.e.

3.2 $$y(t_{i})\approx N_{\theta }^{\;\mathrm{Imp}}(t_{i})=:\;x(t_{i}),$$

where *i* ∈ {1, …, *m*} with *m* being the total number of measurements. Additionally, let us denote another neural network by $N_{\phi }^{\mathrm{Dyn}}$ parameterized by ***ϕ*** that approximates the vector field **g**(·). We connect these two networks by enforcing that the time-evolution of the output of the network $N_{\theta }^{\mathrm{Imp}}$ can be described by $N_{\phi }^{\mathrm{Dyn}}$, i.e.

3.3 $$x(t_{i+1})\approx x(t_{i})+\int_{t_{i}}^{t_{j}}g(x(\tau ))\;d\tau \;\text{and}\;\dot{x}(t_{i})\approx N_{\phi }^{\;\mathrm{Dyn}}(x(t_{i})),$$

where **x**(*t*) is defined in (3.2). As a result, our goal becomes to determine the network parameters {***θ***, ***ϕ***} such that the following loss is minimized:

3.4 $$L=\lambda_{MSE}\cdot L_{MSE}+\lambda_{Integral}\cdot L_{Integral}+\lambda_{Grad}\cdot L_{Grad},$$

where

— $L_{MSE}$ denotes the mean square error of the output of the network $N_{\theta }^{\mathrm{Imp}}$ and the noisy measurements, i.e.
  3.5 $$L_{MSE}\;:=\frac{1}{m}\sum_{i}\|N_{\theta }^{\;\mathrm{Imp}}(t_{i})-y(t_{i}){\|}_{F}^{2},$$
  where $y(t_{i})$ is measurement data. The loss enforces measurement data to be in the vicinity of the output of the implicit network, and $\lambda_{MSE}$ is its weighting parameter.
— The term $L_{Integral}$ links the two networks by comparing the prediction, i.e.
  3.6 $$L_{Integral}\;:=\frac{1}{m-1}\sum_{i}{\left\|x(t_{i+1})-x(t_{i})-\int_{t_{i}}^{t_{i+1}}g(x(\tau ))\;d\tau \right\|}_{F}^{2},$$
  where $x(t_{i})\;:\;=N_{\theta }^{\mathrm{Imp}}(t_{i})$ and the parameter $\lambda_{Integral}$ defines its weight in the total loss.
— The vector field at the output of the implicit network can also be computed directly using automatic differentiation, but it can also be computed using the network $N_{\phi }^{\mathrm{Dyn}}$. The term $L_{Grad}$ penalizes its mismatch as follows:
  3.7 $$L_{Grad}\;:=\frac{1}{m}\sum_{i}\|N_{\phi }^{\;\mathrm{Dyn}}(x(t_{i}))-\dot{x}(t_{i}){\|}_{F}^{2},$$
  and $\lambda_{Grad}$ is its corresponding weighting parameter.

The total loss L can be minimized using a gradient-based optimizer such as Adam [55]. Once the networks are trained and have found their parameters that minimize the loss, we can generate the denoised variables using the implicit network $N_{\theta }^{\mathrm{Imp}}$, and the vector field by the network $N_{\phi }^{\mathrm{Dyn}}$. In the rest of the paper, we denote the proposed methodology by implicit–neural ODEs (in short Imp-NODEs).

## 4. Numerical experiments

We now investigate the performance of the approach discussed in §3 to denoise measurement data and to learn a model for estimating the vector field by means of an example. To that aim, we consider data obtained by solving a differential equation that is then corrupted using additive Gaussian white noise by varying the noise level. For a given percentage, we determine the noise as follows:

$$\nu ∼N(0,\sigma^{2}),\;\text{with}\;\sigma =\frac{\mathrm{Noise}\%}{100}.$$

### 4.1. Training set-up

We have implemented our framework using the deep learning library PyTorch [56] and have optimized both networks simultaneously using the Adam optimizer [55]. We have used torchdiffeq [50], a Python package, to integrate ODEs and to do back-propagation to determine gradients with the default settings. Since at the start of training the parameters of the neural networks are far from the optimized values as they are initialized randomly, it is not required to solve the integral term in (3.6) very accurately. Therefore, we can approximate it using the fourth-order Runge–Kutta (RK4) method at the beginning of the training. Consequently, we can expect to gain computational advantages because the RK4 method requires only four calls of the function defining the vector field. Therefore, we first train using this approximation of the integral for 5000 epochs, followed by training using an adaptive ODE integration scheme for 10 000 epochs. We also make use of a learning scheduler, for which we reduced the learning rate by one-tenth after every 4000 epochs. Furthermore, for the implicit networks, we map the input data to [−1, 1]. Note that, in our experiments, we report the results obtained from one attempt by setting the random seed to 42, except in §4.2.5, where we discuss an ensemble approach. The neural network architecture design and hyper-parameters are discussed in appendix A, and we have run all our experiments on a nvidia P100 GPU.

### 4.2. Cubic damped model

For illustration purposes, we consider a simple damped cubic system, which is described by

4.1 $$\begin{array}{rl} {\dot{x}}_{1}(t) & =-0.1x_{1}{\left(t\right)}^{3}+2.0x_{2}{\left(t\right)}^{3}\\ \mathrm{and}\;\;\;\;\;\;{\dot{x}}_{2}(t) & =-2.0x_{1}{\left(t\right)}^{3}-0.1x_{2}{\left(t\right)}^{3}. \end{array}\}$$

It has been one of the benchmark examples in discovering models using data (e.g. [32,57]), but it is assumed that the dynamics can be given sparsely in a high-dimensional feature dictionary. Here, we do not make such an assumption but instead learn the vector field using a neural network. For this example, we take 2500 data points in the time interval [0, 25] by simulating the model using the initial condition [2, 0]. We add various noise levels to the clean data to obtain noisy measurements synthetically. We, therefore, corrupt the data by adding mean-zero Gaussian white noise with {5%,…,30%} noise.

#### 4.2.1. Training and results

Next, we aim to obtain a denoised signal and a model, defining its vector field using the proposed methodology. Thus, we construct neural networks for the implicit representation and the vector field with the parameters given in table 2.

**Figure 2.**
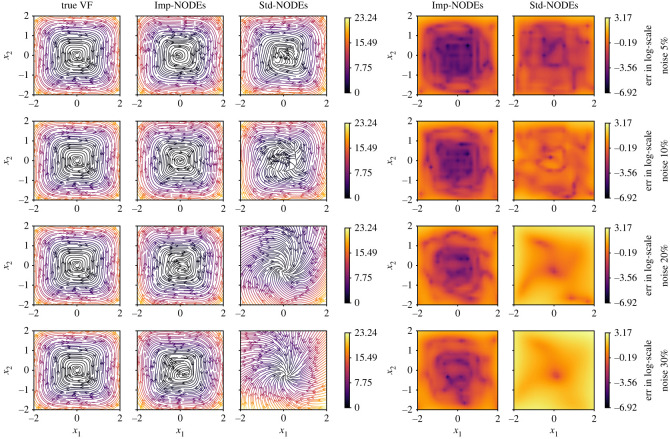
Cubic2D example. A comparison of vector fields of the ground truth and learned models for various noise levels.

To train the implicit network and the neural network for ODEs, we set $\lambda_{Integral}=1.0$ and $\lambda_{Grad}=10^{-2}$ in the loss function (3.4); we choose $\lambda_{MSE}=1.0$ for 5% noise, and $\lambda_{MSE}=0.5$ for {10%,20%} noise, and $\lambda_{MSE}=0.2$ for 30% noise to avoid over-fitting of noisy data for the implicit network. Moreover, to integrate the ODEs, we consider the time span of 10⋅dt with $dt=10^{-2}$. We compare our methodology with the neural ODE framework [50], which also focuses on learning a neural network that defines the underlying vector field. We note that the neural ODE framework does not have any special treatment to handle noise. For this methodology, we train the model for 1000 epochs only, since we shall later illustrate that it is prone to over-fitting when trained longer. Furthermore, it is trained with the same configuration for neural network architectures and using training data as for our approach. Having the trained models, we compare the vector field in the domain [−2, 2] × [−2, 2] by taking 25 points in each direction. We plot the results in figure 2, where the learned models of the vector fields obtained from the proposed method (Imp-NODEs) are compared with neural ODE [50] (Std-NODEs).

**Figure 3.**
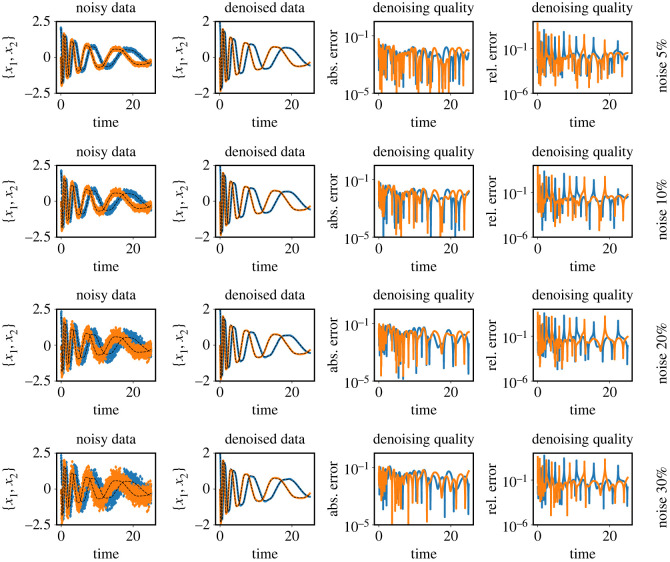
Cubic2D example. The plots show the noisy data, and denoised (recovered) data from the implicit network. The clean (reference) data are indicated using the black dashed lines. The third and fourth columns indicate absolute and relative errors between ground truth and denoised data, respectively.

It is clear from the figures that Imp-NODEs is able to learn the underlying vector field faithfully, whereas Std-NODEs fails to identify the vector fields correctly which becomes particularly evident for higher noise levels. Our approach consists of an implicit network, aiming to generate denoised data in the vicinity of noisy data whose dynamics is defined by a neural network. Thus, we plot the denoised data obtained from the implicit network in figure 3. We note that despite not employing any data-filtering scheme, we can obtain denoised data, close to the ground truth clean data even for a high noise level, which is, otherwise, not possible by employing solely Std-NODEs. We note that the proposed method takes 0.11 s for one epoch, and a similar order of computational time is taken for Std-NODEs.

#### 4.2.2. Hyper-parameter effects on performance of Imp-NODEs

**Figure 4.**
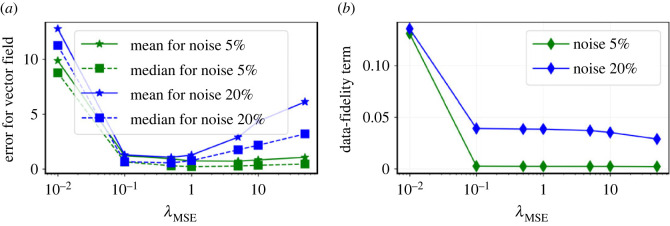
Cubic2D example. (*a*) The effect of the hyper-parameter $\lambda_{MSE}$ on the performance of Imp-NODEs. (*b*) We demonstrate how an *L*-curve study can help to determine a suitable regime for the hyper-parameter $\lambda_{MSE}$. Note that the corner of the *L*-curve is often a good region for $\lambda_{MSE}$, which is also observed in this case.

Next, we study the effect of hyper-parameters on the performance of the proposed methodology. To that end, we first note that for Imp-NODEs, the loss function is given by a weighted sum of three terms; see (3.4). Here, we aim to study the influence of one of the hyper-parameters, namely $\lambda_{MSE}$, on the performance of the learned model using Imp-NODEs, by keeping the other two hyper-parameters fixed. They are set to $\lambda_{Integral}=1.0$ and $\lambda_{Grad}=10^{-2}$, similar to the previous subsection. Recall that $\lambda_{MSE}$ determines how well the given data are approximated using an implicit network. We take the cases of {5%,20%} noise levels. We train different models by varying $\lambda_{MSE}$. We then plot its effect on the performance of the method in figure 4. The figure shows that for low noise levels, the method is robust with respect to the change of the parameter $\lambda_{MSE}$, but it is rather sensitive for high noise levels. This can be explained by the fact that when the data are highly noisy, the implicit neural networks learn the noise by over-fitting, as more weight is given to fitting noisy data. Moreover, when $\lambda_{MSE}$ is very low, then the implicit network does not learn enough information from the data; hence, the underlying vector field cannot be expected to be identified accurately. Therefore, finding a good value of the hyper-parameters is important to obtain a good fit for the model, defining the vector field.

To determine a good region for $\lambda_{MSE}$, we make an attempt and borrow an idea from solving ill-conditioned least-squares problems (e.g. [58]). In light of this, we solve the underlying optimization problem for different values of $\lambda_{MSE}$ and observe the data-fidelity term $L_{MSE}$, given in (3.5). We then plot these quantities, namely $\lambda_{MSE}$ and data-fidelity term, as shown in figure 4*b*. Such a plot often exhibits an *L*-type curve, and a promising region for the value $\lambda_{MSE}$ lies at the corner. These kinds of studies are often carried out to determine hyper-parameters for solving Tikhonov-regularized least-squares problems [58]. It is also what we observe in our case, and such a hyper-parameter search can provide us with a hint about a suitable parameter region.

#### 4.2.3. Longer training effect on Imp-NODEs and Std-NODEs

**Figure 5.**
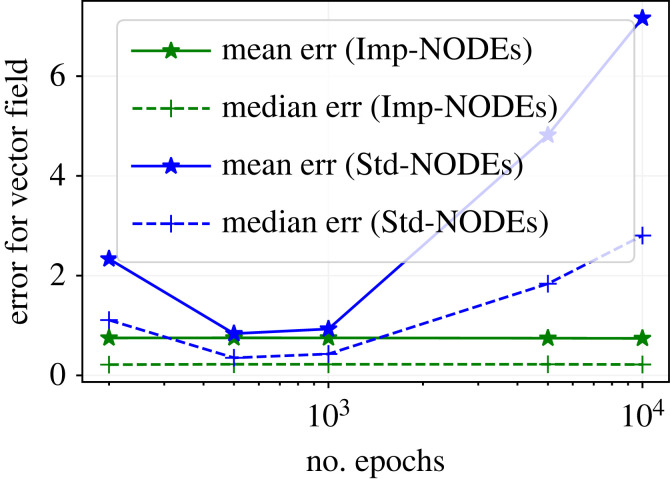
Cubic2D example. The figure illustrates the effect of longer training on the performance of Imp-NODEs and Std-NODEs.

At least in the context of neural networks, it is widely known that longer training with many iterations (or epochs) can over-fit the model, particularly for noisy measurements. Here, we study the effect of longer training on the performance of Imp-NODEs and Std-NODEs. Keeping the same setting as in §4.2.1 and taking data for 20% noise, we learn vector fields using Imp-NODEs and Std-NODEs by varying the number of epochs. We plot the results in figure 5, which shows that Imp-NODEs is quite robust with respect to the number of epochs, and it does not start over-fitting when trained longer. Potential reasoning could be that our approach Imp-NODEs is augmented with an implicit neural network, which can act as an adequate regularizer to avoid over-fitting. By contrast, we observe that Std-NODEs starts learning noise as the training progresses after certain epochs. Hence, early stopping is quite an important factor in having good performance for Std-NODEs.

#### 4.2.4. Employing low-pass filters as a pre-processing step

We have already observed in §4.2.1 that Imp-NODEs is capable of yielding denoised data, which are close to the clean data, without any pre-processing step. However, one might argue that employing classical methods such as low-pass filters can be beneficial, and they provide a computationally cheap yet powerful tool to remove a major part of the noise. This is what we study next—that is, how the performances of Imp-NODEs and Std-NODEs to learn models are affected when a pre-processing step is employed. We have the same setting as in §4.2.1, except that we now employ a low-pass filter to smooth the data. This is achieved by third order digital Butterworth filter with critical frequency 0.1 and is implemented using scipy.^1^

**Figure 6.**
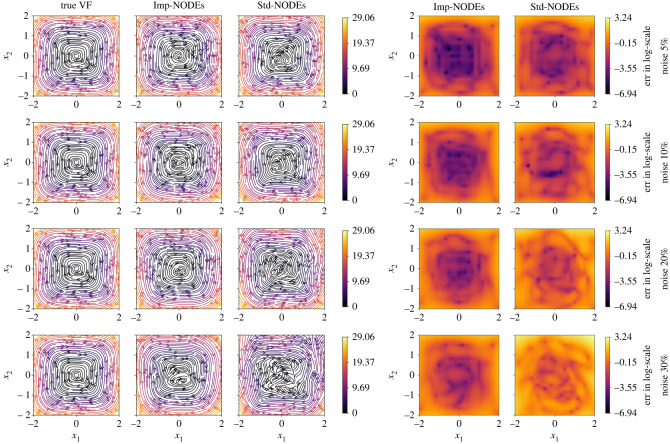
Cubic2D example. Having employed a low-pass filter as a pre-processing step, we here present a comparison of vector fields of the ground truth and learned models for various noise levels.

**Figure 7.**
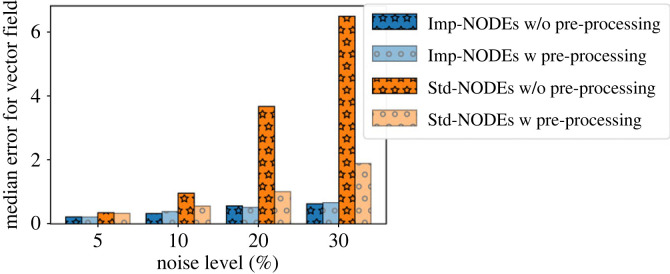
Cubic2D example. The plot shows the effect on employing a pre-processing step using a low-pass filter on both approaches, Imp-NODEs and Std-NODEs. It illustrates that the pre-processing step does not have a major impact on the performance of Imp-NODEs, in contrast to Std-NODEs.

We present the quality of the learned vector fields using Imp-NODEs and Std-NODEs in figure 6. We note that the performance of Std-NODEs is only slightly affected, as compared to the case when no pre-processing step was employed (compare figures 2 and 6). On the other hand, for Std-NODEs, we observe a substantial improvement, especially for high noise levels. A reason is that Std-NODEs does not have an inherent capability of handling noise; hence, any filtering approach would be highly beneficial, in contrast to our approach Imp-NODEs, which implicitly aims to yield denoised data as well. We explicitly report this behaviour by means of a bar plot; see figure 7. Despite improving the performance of Std-NODEs by means of pre-precessing, our approach Imp-NODEs still outperforms Std-NODEs in terms of the quality of the learned models for the vector field. Furthermore, we note that such filtering approaches are not straightforward to employ, especially in the case of irregular data. In those cases, employing our approach, namely Imp-NODEs, could be beneficial since it does not require any pre-processing step yet yields good models and denoised data.

#### 4.2.5. An ensemble approach to improve performance

Ensemble approaches are widely employed machine learning techniques to improve model predictions. The main principle is to combine predictions of many possible independent models, for example, by taking an average. Several methods exist in this direction, such as bragging, bagging and boosting (e.g. [2–4,6,59]). In this work, we take inspiration from bagging and propose the following to obtain an ensemble of models for predicting the vector field.

In bagging, data bootstraps are often used with replacements, followed by learning a model for an ensemble. In our framework, the data are very limited, thus focusing on using all of them in some form. However, we require to build an ensemble of independent models. For this purpose, we propose modifying the terms (3.5)–(3.7) using a weighting vector *ω* as follows:

4.2*a* $$\;L_{MSE}^{\omega }=\frac{1}{m}\sum_{i}\omega_{i}^{2}\|N_{\theta }^{\;I}(t_{i})-y(t_{i}){\|}_{F}^{2},$$

4.2*b* $$\;L_{Integral}^{\omega }=\frac{1}{m}\sum_{i}\omega_{i}^{2}{\left\|x(t_{\;j})-x(t_{i})-\int_{t_{i}}^{t_{j}}g(x(\tau ))\;d\tau \right\|}_{F}^{2},$$

**Figure 8.**
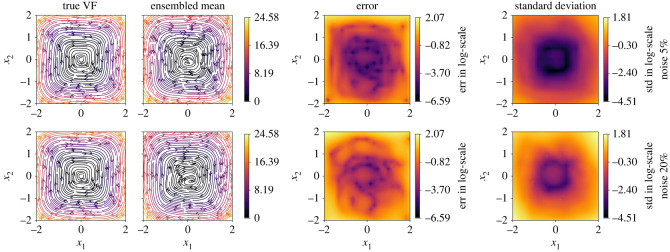
Cubic2D example. The figure demonstrates the performance of the ensemble approach combined with Imp-NODEs. We constructed an ensemble of 20 models and have taken the mean of these to predict the vector field.

4.2*c* $$\mathrm{and}\;\;\;\;\;\;L_{Grad}^{\omega }=\frac{1}{m}\sum_{i}\omega_{i}^{2}\|N_{\phi }^{\;Dyn}(x(t_{i}))-\dot{x}(t_{i}){\|}_{F}^{2}$$

where *ω*_*i*_ is the *i*th entry of *ω* and is sampled randomly from a uniform distribution between [0, 1]. Using the modified loss terms as above, we can define a new weighted total loss as in (3.4). We can expect a different solution for every random vector *ω*, as the underlying optimization problem is highly nonlinear and non-convex. Moreover, a physical interpretation of *ω* or *ω*_*i*_, in the context of the classical bragging philosophy, can be given as follows: it defines a probability of drawing the sample $y_{i}$ (or **x**_*i*_). Consequently, we can obtain an ensemble of models by randomly selecting *ω*. For Imp-NODEs, we build 20 models to predict the vector field for 5% and 20% noise levels and plot the mean of the ensemble models in figure 8. We also show the standard deviation among these 20 models. These figures indicate that we have a good approximation of the vector field in the region of the collected data and can estimate the confidence by means of the obtained standard deviation. To further quantify the performance of the ensemble approach, in table 1 we note the mean and median errors of the vector fields of the mean-ensemble model, the best and worst models among the 20 trained models. Interestingly, we find that the mean-ensemble model can even outperform the best-obtained model with a single attempt. This illustrates the powerful capability of an ensemble approach. However, we note that ensemble approaches come with computational disadvantages, as we are not only required to train several models, but during the inference, we also need to make use of those many models to take an average of them.

**Table 1.** Cubic2D example. A comparison of the mean-ensemble, the best and the worst models is presented by comparing the mean and median of the error of the vector field. It shows that the mean-ensemble model can outperform the best model obtained using a single attempt. The best performing model using mean and median measures is highlighed in bold.

model	5% noise		20% noise	
	mean	median	mean	median
ensemble model	0.724	**0.191**	**1.072**	**0.503**
best model	**0.700**	0.304	1.158	0.697
worst model	0.924	0.282	2.117	1.321

## 5. Second-order neural ODEs for noisy data

Several dynamics observed in engineering processes, particularly in electrical and mechanical systems, are of second order, which can be given as follows:

5.1 $$\ddot{x}(t)=g(x(t),\dot{x}(t)),$$

where $\dot{x}(t)$ and $\ddot{x}(t)$ denote the first and second derivatives of **x**(*t*) with respect to *t*, respectively. As discussed in [60], it is advantageous to consider the companion first-order system of (5.1) which is as follows:

5.2 $$\left[\begin{array}{c} {\dot{z}}_{1}(t)\\ {\dot{z}}_{2}(t) \end{array}\right]=\left[\begin{array}{c} g(z_{2}(t),z_{1}(t))\\ z_{1}(t) \end{array}\right],$$

where $z_{1}(t)=\dot{x}(t)$ and $z_{2}(t)=x(t)$, and it inherently preserves the second-order behaviour. The above system can be seen as a first-order system with a constraint. The method proposed in the previous section can be readily applied to learn second-order neural ODEs for noisy measurements by incorporating implicit networks.

### 5.1. Numerical example: pendulum dynamics

To illustrate learning second-order dynamics, we consider the nonlinear pendulum model

5.3 $$\ddot{x}(t)=-\sin(x(t))-0.05\cdot \dot{x}(t).$$

We collect data using the initial condition $\left[\dot{x}(t),x(t)]=[-0.5,2.0\right]$ with time steps of dt=0.05, which is then corrupted by adding Gaussian white noise of {5%,10%,20%,30%} noise levels. Here, as well, we do not apply any pre-processing step to observe the performance of the proposed methodology without any pre-processing. By imposing the second-order structure, we employ the proposed scheme by combining an implicit network and neural ODEs. We train the networks with parameters $\lambda_{Integral}=1.0$, $\lambda_{Grad}=10^{-2}$, and $\lambda_{MSE}=1.0$ in (3.4) for 5% noise, and $\lambda_{MSE}=0.5$ for 10% and 20% noise, and $\lambda_{MSE}=0.1$ for 30% noise to avoid over-fitting. We also use an early stopping for standard neural ODEs to avoid over-fitting, as discussed in §4.2.1. We train it for 200 epochs. For numerical integration, we take a time span of 5⋅dt.

**Figure 9.**
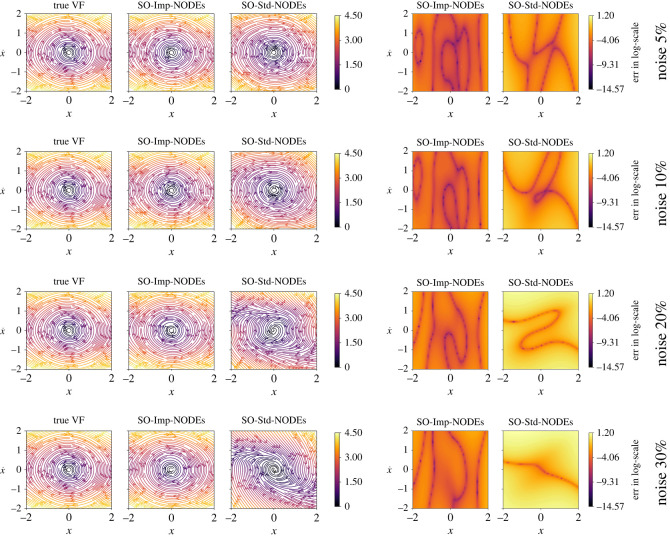
Pendulum example. A comparison of the learned vector fields for second-order dynamical models using the proposed methodology and SO-Std-NODEs for various noise levels. It illustrates the robustness of the proposed approach with respect to various noise levels.

**Figure 10.**
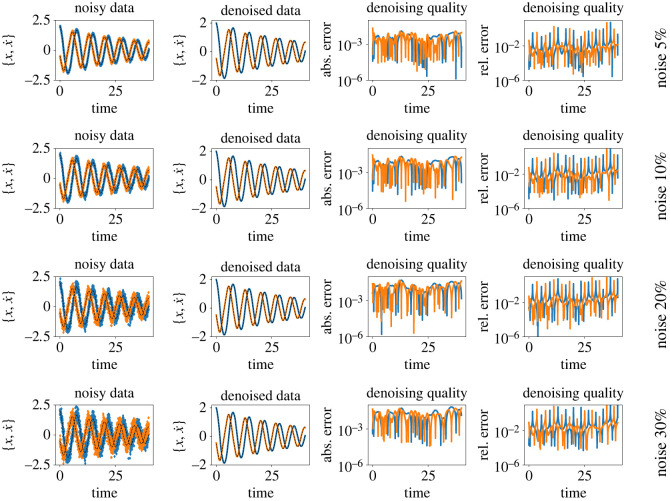
Pendulum example. The figure shows the noisy measurements, and denoised data obtained from the implicit network. The black dashed lines show the ground truth reference. The third and fourth columns indicate absolute and relative errors between ground truth and denoised data, respectively.

We compare our results with neural ODEs for second-order systems, the approach proposed in [60]; we denote it by SO-Std-NODEs. We plot the learned vector field from both methods in figure 9, where we see a better performance for the proposed method than SO-Std-NODEs. It is particularly apparent for more significant noise, where SO-Std-NODEs fails to capture the vector field (see figure 9 third and fourth rows). Moreover, in figure 10, we plot the denoised data, which is the output of the trained implicit network, indicating the faithful recovery of the data without performing any prior pre-processing step.

## 6. Data at irregular sampling

Lastly, we illustrate the ready applicability of the proposed method (Imp-NODEs) when the data are collected at an irregular time grid, especially when dependent variables are not even measured in the same time frame. This is of particular interest in medical applications, where data often come at quite irregular time intervals or when the sources of information are different.

We here present the framework for two-dimensional problems; however, it readily extends to arbitrary dimensional dynamics. Let us consider a dynamical model as follows:

6.1 $$\dot{x}(t)=g(x(t)),$$

where $x=[x_{1},x_{2}]\in R^{2}$. Next, assume that the variable *x*_1_ is measured on the time grid $T_{1}=\{t_{1}^{\left(1\right)},\ldots ,t_{n}^{\left(1\right)}\}$, whereas the variable *x*_2_ is collected on the time grid $T_{2}=\{t_{1}^{\left(2\right)},\ldots ,t_{m}^{\left(2\right)}\}$ with *T*_1_ ≠ *T*_2_. To learn a model for the vector field representing the dynamics for **x** using measurements at an irregular time grid, we construct an implicit representation for **x** so that both variables can be estimated on the same time grid (let us denote it by *T* = {*t*_1_, …, *t*_*p*_}) but with a constraint using measurements. Assume the implicit network and neural ODE defining the vector field are denoted by $N_{\theta }^{\mathrm{Imp}}$ and $N_{\phi }^{\mathrm{Dyn}}$. To train the network, we define the following loss function:

$$\begin{array}{rl} & \lambda_{MSE}\left(\frac{1}{n}\sum_{i}\|{\left[N_{\theta }^{\;\mathrm{Imp}}(t_{i}^{\left(1\right)})\right]}_{1}-x_{1}(t_{i}^{\left(1\right)}){\|}_{F}+\frac{1}{m}\sum_{i}\|{\left[N_{\theta }^{\;\mathrm{Imp}}(t_{i}^{\left(2\right)})\right]}_{2}-x_{2}(t_{i}^{\left(2\right)}){\|}_{F}\right)\\ & \;+\lambda_{Grad}\left(\frac{1}{p}\sum_{i}{\left\|N_{\phi }^{\;\mathrm{Dyn}}(N_{\theta }^{\;\mathrm{Imp}}(t_{i}))-\frac{d}{dt}N_{\theta }^{\;\mathrm{Imp}}(t_{i})\right\|}_{F}\right)\\ & \;+\lambda_{Integral}\left(\frac{1}{p}\sum_{i}{\left\|(N_{\theta }^{\;\mathrm{Imp}}(t_{i+1})-N_{\theta }^{\;\mathrm{Imp}}(t_{i}))-\int_{t_{i}}^{t_{i+1}}N_{\phi }^{\;\mathrm{Dyn}}(N_{\theta }^{\;\mathrm{Imp}}(\tau ))\;d\tau \right\|}_{F}\right), \end{array}$$

where [ · ]_*k*_ denotes the *k*th element.

### 6.1. Numerical example: linear 2D

We illustrate the considered scenario using a linear 2D example given by

$${\dot{x}}_{1}(t)=0.1\cdot x_{1}(t)+2.0\cdot x_{2}(t)$$

and

$${\dot{x}}_{2}(t)=2.0\cdot x_{1}(t)-0.1\cdot x_{2}(t).$$

**Figure 11.**
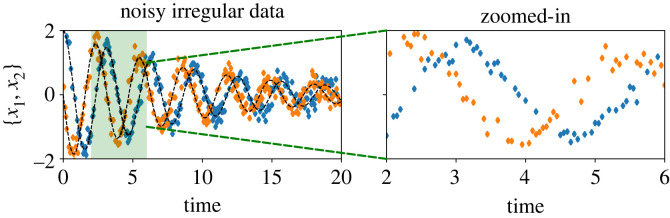
Linear 2D example. An illustration of collected noisy and irregular data. In the zoomed-in plot, it is clearly visible that the variables *x*_1_ and *x*_2_ are not collected at the same time frame.

We collect data using an initial condition [*x*_1_, *x*_2_] = [2, 0] with a time step dt=0.05 in the time interval [0, 20]. We randomly collect 60% independent samples for the first and second dependent variables, followed by corrupting them using Gaussian white noise for {5%,10%,20%,30%} noise levels. Consequently, we obtain the data, which are not only noisy but irregular as well, as shown in figure 11. Furthermore, we take the time grid for prediction of the output of the implicit network as the uniform grid with dt=0.05 for the time interval [0, 20] so that it can be fed to evaluate the integral terms. For learning models for the vector field, we set $\lambda_{Integral}=1.0$, $\lambda_{Grad}=10^{-2}$, and $\lambda_{MSE}=1.0$ for 5% noise level, $\lambda_{MSE}=0.5$ for 10% noise level, and $\lambda_{MSE}=0.2$ for {20%,30%} noise levels. For time integration, we consider the time span 5⋅dt.

**Figure 12.**
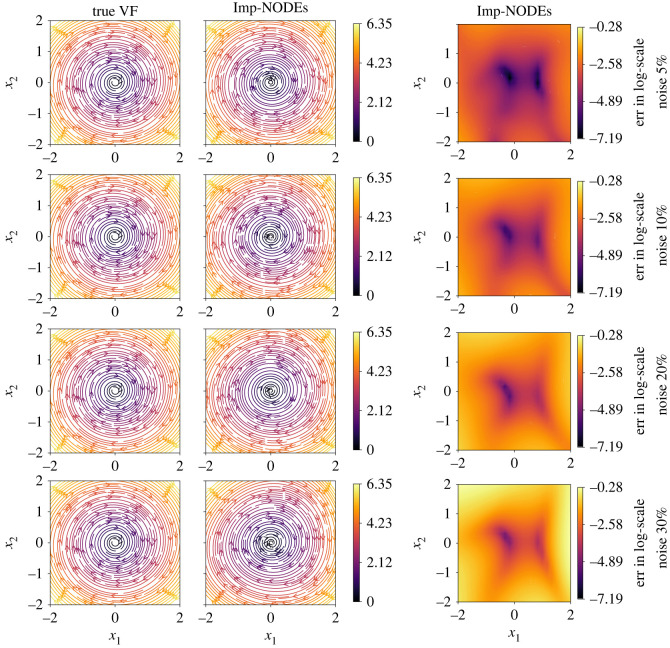
Linear 2D example. A comparison of the learned vector fields for second-order dynamical models using the proposed methodology and Std-NODEs for various noise levels. It illustrates the capability of the proposed method to learn dynamic models from highly irregular data and its robustness with respect to various noise levels.

**Figure 13.**
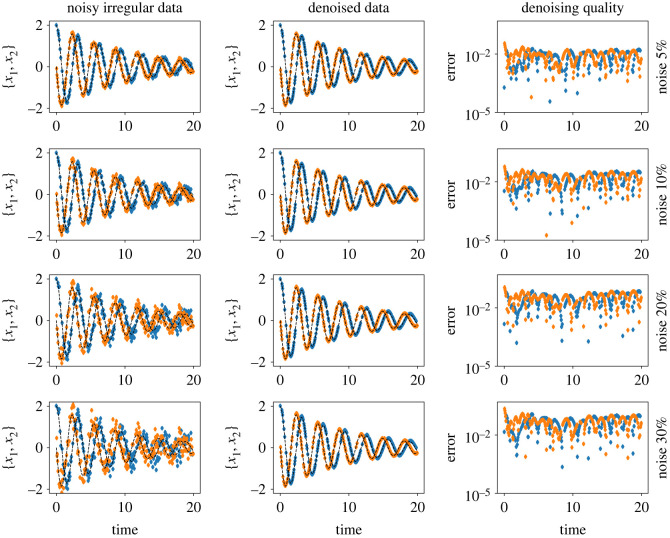
Linear 2D example. The figure shows the ability to recover the clean data by means of the implicit network, even for irregular data.

We show the estimates of the learned vector fields using the proposed methodology and compare them with the ground truth in figure 12, illustrating faithful capturing of the dynamics. Moreover, we can recover the clean signal without any prior information about the noise and any pre-processing of the data, even for irregular data, as shown in figure 13.

## 7. Discussion and conclusion

This work has presented a new approach for learning dynamical models from noisy time-series data and for obtaining denoised data. Our framework blends the universal approximation capabilities of deep neural networks with neural ODEs. The proposed scheme involves two networks to learn (approximately) an implicit representation of the measurement data and of the vector field. These networks are combined by enforcing that an ODE can explain the dynamics of the output of the implicit network. We also discussed its extension to second-order neural ODEs to learn second-order dynamical models using corrupted data. Furthermore, we have presented that the proposed approach can readily handle arbitrarily sampled points in time. The dependent variables need not be collected at the same time grid. This is possible because of the construction of an implicit representation of the data in our framework that does not require data to be at a particular grid. We also discussed an ensemble approach, inspired by bragging, to improve the quality of the models by taking an average of an ensemble of models. We have also discussed a scheme based on an *L*-curve analysis to determine a good regime for hyper-parameters.

In the future, we will focus on using the encoder–decoder framework combined with an implicit network to learn latent ODEs and explain even richer dynamics. Moreover, when the data are high-dimensional (e.g. coming from partial differential equations), applying neural ODEs becomes computationally intractable. However, it is known that the dynamics often lie in a low-dimensional manifold. Therefore, in our future work, we aim to use the concept of low-dimensional embedding to make learning computationally more efficient for high-dimensional data. Furthermore, it would be interesting to use expert knowledge and physical laws to have physics-constrained neural ODEs so that the generalizability and extrapolation capabilities of models can be further improved.

## Figures and Tables

**Figure 14.**
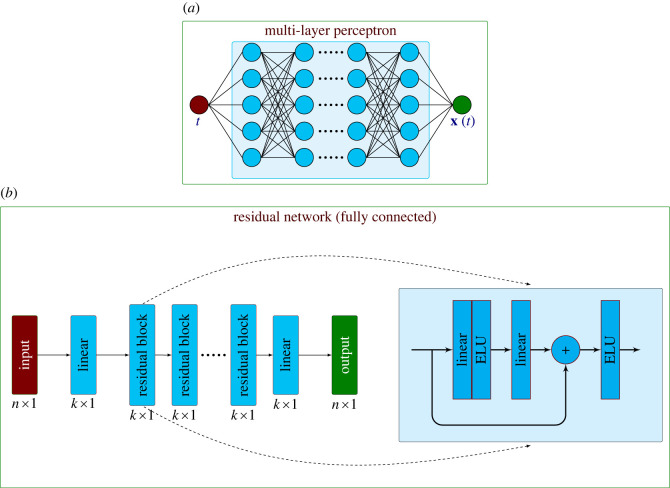
The figure shows two potential simple architectures that can be used to learn either implicit representation or to approximate the underlying vector field. (*a*) A simple multi-layer perceptron and (*b*) a residual-type network but fully connected.

## Data Availability

Data and relevant code for this research work are stored in GitLab: https://gitlab.mpi-magdeburg.mpg.de/goyalp/implicit_neuralodes and have been archived within the Zenodo Repository: https://doi.org/10.5281/zenodo.8063848 [61].

## Footnotes

1 The command scipy.single.butter(N=3, Wn=0.1, btype = ‘lowpass’).

## Appendix A. Suitable architectures and chosen hyper-parameter

Here, we briefly discuss neural network architectures suitable for our proposed approach. We require two neural networks for our framework, one for learning the implicit representation $N_{\theta }^{I}$ and the second one $N_{\theta }^{\mathrm{Dyn}}$ is to learn the vector field. For implicit representation, we use a fully connected multi-layer perceptron (MLP) as depicted in figure 14*a* with periodic activation functions (e.g. sin) [62] which has shown its ability to capture finely detailed features as well as the gradients of a function. To approximate the vector field, we consider a simple residual-type network as illustrated in figure 14*b* with *exponential linear unit* (ELU) as an activation function [63]. We choose ELU as the activation function since it is continuous and differentiable and resembles a widely used activation function, namely rectified linear unit (ReLU). For the considered examples in the paper, we report the architecture designs (e.g. numbers of neural and layers) in table 2.

**Table 2.** The table shows the information about network architectures and learning rates.

example	networks	neurons	layers or residual blocks	learning rates
cubic oscillator	for implicit representation	20	4	10^−3^
	for approximating vector field	20	4	10^−3^
pendulum	for implicit representation	32	4	5 × 10^−4^
	for approximating vector field	20	4	10^−3^
linear example	for implicit representation	20	3	5 × 10^−4^
	for approximating vector field	20	2	10^−3^

## References

[1] Rudy SH, Kutz JN, Brunton SL. 2019 Deep learning of dynamics and signal-noise decomposition with time-stepping constraints. J. Comput. Phys. **396**, 483-506. (10.1016/j.jcp.2019.06.056)

[2] Schapire RE. 1990 The strength of weak learnability. Mach. Learn. **5**, 197-227. (10.1007/BF00116037)

[3] Breiman L. 1996 Bagging predictors. Mach. Learn. **24**, 123-140. (10.1007/BF00058655)

[4] Bühlmann PL. 2003 Bagging, subagging and bragging for improving some prediction algorithms. In Recent advances and trends in nonparametric statistics (eds MG Akritas, DN Politis), pp. 19-34. Amsterdam, The Netherlands: Elsevier.

[5] Polikar R. 2006 Ensemble based systems in decision making. IEEE Circuits Syst. Mag. **6**, 21-45. (10.1109/MCAS.2006.1688199)

[6] James G, Witten D, Hastie T, Tibshirani R. 2013 An introduction to statistical learning, vol. 112. New York, NY: Springer.

[7] Juang JN. 1994 Applied system identification. Upper Saddle River, NJ: Prentice-Hall.

[8] Ljung L. 1999 System identification—theory for the user, 2nd edn. Upper Saddle River, NJ: Prentice-Hall.

[9] Billings SA. 2013 Nonlinear system identification: NARMAX methods in the time, frequency, and spatio-temporal domains. Hoboken, NJ: John Wiley & Sons.

[10] Ho B, Kálmán RE. 1966 Effective construction of linear state-variable models from input/output functions. Automatisierungstechnik **14**, 545-548. (10.1524/auto.1966.14.112.545)

[11] Juang JN, Pappa RS. 1985 An eigensystem realization algorithm for modal parameter identification and model reduction. J. Guid. Control Dyn. **8**, 620-627. (10.2514/3.20031)

[12] Longman RW, Juang JN. 1989 Recursive form of the eigensystem realization algorithm for system identification. J. Guid. Control Dyn. **12**, 647-652. (10.2514/3.20458)

[13] Juang JN, Phan M, Horta LG, Longman RW. 1993 Identification of observer/Kalman filter Markov parameters: theory and experiments. J. Guid. Control Dyn. **16**, 320-329. (10.2514/3.21006)

[14] Phan M, Horta LG, Juang JN, Longman RW. 1993 Linear system identification via an asymptotically stable observer. J. Optim. Theory Appl. **79**, 59-86. (10.1007/BF00941887)

[15] Phan M, Juang JN, Longman RW. 1992 Identification of linear multivariable systems by identification of observers with assigned real eigenvalues. J. Astronaut. Sci. **40**, 261-279.

[16] Kalman RE. 1960 A new approach to linear filtering and prediction problems. J. Fluid Eng. **82**, 35-45. (10.1115/1.3662552)

[17] Schmid PJ. 2010 Dynamic mode decomposition of numerical and experimental data. J. Fluid Mech. **656**, 5-28. (10.1017/S0022112010001217)

[18] Tu JH, Rowley CW, Luchtenburg DM, Brunton SL, Kutz JN. 2014 On dynamic mode decomposition: theory and applications. J. Comput. Dyn. **1**, 391-421. (10.3934/jcd.2014.1.391)

[19] Kevrekidis IG, Gear CW, Hyman JM, Kevrekidis PG, Runborg O, Theodoropoulos C. 2003 Equation-free, coarse-grained multiscale computation: enabling macroscopic simulators to perform system-level analysis. Commun. Math. Sci. **1**, 715-762.

[20] Voss HU, Kolodner P, Abel M, Kurths J. 1999 Amplitude equations from spatiotemporal binary-fluid convection data. Phys. Rev. Lett. **83**, 3422-3425. (10.1103/PhysRevLett.83.3422)

[21] Ye H, Beamish RJ, Glaser SM, Grant SC, Hsieh CH, Richards LJ, Schnute JT, Sugihara G. 2015 Equation-free mechanistic ecosystem forecasting using empirical dynamic modeling. Proc. Natl Acad. Sci. USA **112**, E1569-E1576. (10.1073/pnas.1417063112) 25733874 PMC4386326

[22] Schmidt MD, Vallabhajosyula RR, Jenkins JW, Hood JE, Soni AS, Wikswo JP, Lipson H. 2011 Automated refinement and inference of analytical models for metabolic networks. Phys. Biol. **8**, 055011. (10.1088/1478-3975/8/5/055011) 21832805 PMC4109817

[23] Daniels BC, Nemenman I. 2015 Automated adaptive inference of phenomenological dynamical models. Nat. Commun. **6**, 8133. (10.1038/ncomms9133) 26293508 PMC4560822

[24] Daniels BC, Nemenman I. 2015 Efficient inference of parsimonious phenomenological models of cellular dynamics using S-systems and alternating regression. PLoS ONE **10**, e0119821. (10.1371/journal.pone.0119821) 25806510 PMC4373916

[25] Bongard J, Lipson H. 2007 Automated reverse engineering of nonlinear dynamical systems. Proc. Natl Acad. Sci. USA **104**, 9943-9948. (10.1073/pnas.0609476104) 17553966 PMC1891254

[26] Schmidt M, Lipson H. 2009 Distilling free-form natural laws from experimental data. Science **324**, 81-85. (10.1126/science.1165893) 19342586

[27] Brunton SL, Proctor JL, Kutz JN. 2016 Sparse identification of nonlinear dynamics with control (SINDYc). IFAC-PapersOnLine **49**, 710-715. (10.1016/j.ifacol.2016.10.249)

[28] Mangan NM, Brunton SL, Proctor JL, Kutz JN. 2016 Inferring biological networks by sparse identification of nonlinear dynamics. IEEE Trans. Mol. Biol. Multi-Scale Commun. **2**, 52-63. (10.1109/TMBMC.2016.2633265)

[29] Tran G, Ward R. 2017 Exact recovery of chaotic systems from highly corrupted data. Multiscale Model. Simul. **15**, 1108-1129. (10.1137/16M1086637)

[30] Schaeffer H, Tran G, Ward R, Zhang L. 2020 Extracting structured dynamical systems using sparse optimization with very few samples. Multiscale Model. Simul. **18**, 1435-1461. (10.1137/18M1194730)

[31] Mangan NM, Kutz JN, Brunton SL, Proctor JL. 2017 Model selection for dynamical systems via sparse regression and information criteria. Proc. R. Soc. A **473**, 20170009. (10.1098/rspa.2017.0009) 28878554 PMC5582175

[32] Goyal P, Benner P. 2022 Discovery of nonlinear dynamical systems using a Runge-Kutta inspired dictionary-based sparse regression approach. Proc. R. Soc. A **478**, 20210883. (10.1098/rspa.2021.0883) 35756880 PMC9215218

[33] Raissi M, Karniadakis GE. 2018 Hidden physics models: machine learning of nonlinear partial differential equations. J. Comput. Phys. **357**, 125-141. (10.1016/j.jcp.2017.11.039)

[34] Chen S, Billings SA, Grant P. 1990 Non-linear system identification using neural networks. Int. J. Control **51**, 1191-1214. (10.1080/00207179008934126)

[35] Rico-Martinez R, Kevrekidis IG. 1993 Continuous time modeling of nonlinear systems: a neural network-based approach. In IEEE Int. Conf. on Neural Networks, San Francisco, CA, USA, 28 March–1 April 1993, pp. 1522-1525. (10.1109/ICNN.1993.298782)

[36] Gonzalez-Garcia R, Rico-Martinez R, Kevrekidis I. 1998 Identification of distributed parameter systems: a neural net based approach. Comput. Chem. Eng. **22**, S965-S968.

[37] Milano M, Koumoutsakos P. 2002 Neural network modeling for near wall turbulent flow. J. Comput. Phys. **182**, 1-26. (10.1006/jcph.2002.7146)

[38] Lu Z, Hunt BR, Ott E. 2018 Attractor reconstruction by machine learning. Chaos **28**, 061104. (10.1063/1.5039508) 29960382

[39] Pan S, Duraisamy K. 2018 Long-time predictive modeling of nonlinear dynamical systems using neural networks. Complexity **2018**, 4801012. (10.1155/2018/4801012)

[40] Pathak J, Lu Z, Hunt BR, Girvan M, Ott E. 2017 Using machine learning to replicate chaotic attractors and calculate Lyapunov exponents from data. Chaos **27**, 121102. (10.1063/1.5010300) 29289043

[41] Pathak J, Wikner A, Fussell R, Chandra S, Hunt BR, Girvan M, Ott E. 2018 Hybrid forecasting of chaotic processes: using machine learning in conjunction with a knowledge-based model. Chaos **28**, 041101. (10.1063/1.5028373) 31906641

[42] Vlachas PR, Byeon W, Wan ZY, Sapsis TP, Koumoutsakos P. 2018 Data-driven forecasting of high-dimensional chaotic systems with long short-term memory networks. Proc. R. Soc. A **474**, 20170844. (10.1098/rspa.2017.0844) 29887750 PMC5990702

[43] Lusch B, Kutz JN, Brunton SL. 2018 Deep learning for universal linear embeddings of nonlinear dynamics. Nat. Commun. **9**, 4950. (10.1038/s41467-018-07210-0) 30470743 PMC6251871

[44] Takeishi N, Kawahara Y, Yairi T. 2017 Learning Koopman invariant subspaces for dynamic mode decomposition. In Advances in neural information processing systems, vol. 30 (eds I Guyon, U Von Luxburg, S Bengio, H Wallach, R Fergus, S Vishwanathan, R Garnett), pp. 1130-1140. New York, NY: Curran Associates.

[45] Yeung E, Kundu S, Hodas N. 2019 Learning deep neural network representations for Koopman operators of nonlinear dynamical systems. In *American Control Conf. (ACC), Philadelphia, PA, USA, 10–12 July 2019*, pp. 4832–4839. (10.23919/ACC.2019.8815339)

[46] Champion K, Lusch B, Kutz JN, Brunton SL. 2019 Data-driven discovery of coordinates and governing equations. Proc. Natl Acad. Sci. USA **116**, 22 445-22 451. (10.1073/pnas.1906995116) PMC6842598 31636218

[47] Raissi M, Perdikaris P, Karniadakis GE. 2018 Multistep neural networks for data-driven discovery of nonlinear dynamical systems. (https://arxiv.org/abs/1801.01236) 10.1098/rspa.2016.0751 PMC5332612 28293137

[48] Raissi M, Perdikaris P, Karniadakis GE. 2019 Physics-informed neural networks: a deep learning framework for solving forward and inverse problems involving nonlinear partial differential equations. J. Comput. Phys. **378**, 686-707. (10.1016/j.jcp.2018.10.045)

[49] Raissi M, Yazdani A, Karniadakis GE. 2020 Hidden fluid mechanics: learning velocity and pressure fields from flow visualizations. Science **367**, 1026-1030. (10.1126/science.aaw4741) 32001523 PMC7219083

[50] Chen RT, Rubanova Y, Bettencourt J, Duvenaud DK. 2018 Neural ordinary differential equations. In Advances in neural information processing systems, vol. 31 (eds S Bengio, H Wallach, H Larochelle, K Grauman, N Cesa-Bianchi, R Garnett), pp. 6571-6583. New York, NY: Curran Associates.

[51] Messenger DA, Bortz DM. 2021 Weak SINDy: galerkin-based data-driven model selection. Multiscale Model. Simul. **19**, 1474-1497. (10.1137/20M1343166) 38239761 PMC10795802

[52] Fasel U, Kutz JN, Brunton BW, Brunton SL. 2022 Ensemble-SINDy: robust sparse model discovery in the low-data, high-noise limit, with active learning and control. Proc. R. Soc. A **478**, 20210904. (10.1098/rspa.2021.0904) 35450025 PMC9006119

[53] Rubanova Y, Chen RTQ, Duvenaud DK. 2019 Latent ordinary differential equations for irregularly-sampled time series. In Advances in neural information processing systems, vol. 32 (eds H Wallach, H Larochelle, A Beygelzimer, F d Alchè-Buc, E Fox, R Garnett), pp. 5321-5331. New York, NY: Curran Associates.

[54] Bhouri MA, Perdikaris P. 2022 Gaussian processes meet NeuralODEs: a Bayesian framework for learning the dynamics of partially observed systems from scarce and noisy data. Phil. Trans. R. Soc. A **380**, 20210201. (10.1098/rsta.2021.0201) 35719075

[55] Kingma DP, Ba J. 2014 Adam: a method for stochastic optimization. (https://arxiv.org/abs/1412.6980)

[56] Paszke A et al. 2019 Pytorch: an imperative style, high-performance deep learning library. (https://arxiv.org/abs/1912.01703)

[57] Brunton SL, Proctor JL, Kutz JN. 2016 Discovering governing equations from data by sparse identification of nonlinear dynamical systems. Proc. Natl Acad. Sci. USA **113**, 3932-3937. (10.1073/pnas.1517384113) 27035946 PMC4839439

[58] Lawson CL, Hanson RJ. 1995 Solving least squares problems. Philadelphia, PA: SIAM.

[59] Bühlmann P. 2012 Bagging, boosting and ensemble methods. In Handbook of computational statistics: concepts and methods (eds JE Gental, WK Härdle, Y Mori), pp. 985-1022. Berlin, Germany: Springer. (10.1007/978-3-642-21551-3_33)

[60] Norcliffe A, Bodnar C, Day B, Simidjievski N, Liò P. 2020 On second order behaviour in augmented neural ODEs. In Advances in neural information processing systems, vol. 33 (eds H Larochelle, M Ranzato, R Hadsell, MF Balcan, H Lin), pp. 5911-5921. New York, NY: Curran Associates.

[61] Goyal P, Benner P. 2023 Code for: Neural ODEs with irregular and noisy data. Zenodo. (10.5281/zenodo.8063848) PMC10354476 37476515

[62] Sitzmann V, Martel J, Bergman A, Lindell D, Wetzstein G. 2020 Implicit neural representations with periodic activation functions. In Advances in neural information processing systems, vol. 33 (eds H Larochelle, M Larochelle, R Hadsell, MF Balcan, H Lin), pp. 7462-74673. New York, NY: Curran Associates.

[63] Clevert DA, Unterthiner T, Hochreiter S. 2015 Fast and accurate deep network learning by exponential linear units (ELUs). (https://arxiv.org/abs/1511.07289)

